# A Novel and Cost-Effective CsVO_3_ Quantum Dots for Optoelectronic and Display Applications

**DOI:** 10.3390/nano12162864

**Published:** 2022-08-19

**Authors:** Ganji Seeta Rama Raju, Ganji Lakshmi Varaprasad, Jeong-Hwan Lee, Jin Young Park, Nilesh R. Chodankar, Kugalur Shanmugam Ranjith, Eluri Pavitra, Yun Suk Huh, Young-Kyu Han

**Affiliations:** 1 Department of Energy and Materials Engineering, Dongguk University-Seoul, Seoul 04620, Korea; 2 Department of Biological Engineering, Biohybrid Systems Research Center (BSRC), Inha University, Incheon 22212, Korea; 3 Department of Materials Science and Engineering, Inha University, Incheon 22212, Korea; 4 Department of Electrical, Electronics and Software Engineering, Pukyong National University, Yongdang Campus, Busan 48547, Korea

**Keywords:** CsVO_3_ quantum dots, hotplate synthesis, quantum confinement effect, tunable emissions

## Abstract

Quantum dots (QDs) have an unparalleled ability to mimic true colors due to their size-tunable optical and electronic properties, which make them the most promising nanoparticles in various fields. Currently, the majority of QDs available in the market are cadmium, indium, and lead-based materials but the toxicity and unstable nature of these QDs restricts their industrial and practical applications. To avoid using heavy metal ions, especially cadmium, the current research is focused on the fabrication of perovskite and vanadate QDs. Herein, we report the facile synthesis of a novel and cost-effective CsVO_3_ QDs for the first time. The sizes of the CsVO_3_ QDs produced were tuned from 2 to 10 nm by varying the reaction temperature from 140 to 190 °C. On increasing QD size, a continuous red shift was observed in absorption and emission spectra, signifying the presence of quantum confinement. In addition, along with CsVO_3_ QDs, the CsVO_3_ nanosheets self-assembled microflower-like particles were found as residue after the centrifugation; the X-ray diffraction indicated an orthorhombic structure. Under 365 nm excitation, these CsVO_3_ microflower-like particles exhibited broad emission with CIE coordinates in the white emission region. The acquired results suggest that CsVO_3_ QDs may represent a new class of cadmium-free materials for optoelectronic and biomedical applications.

## 1. Introduction

Research on semiconductor nanocrystals, also called colloidal quantum dots (QDs), has dominated the field of nanoscience because of their ability to tune optical and electronic properties through size control [1,2,3]. Ever since, Colvin et al. [4] described the CdSe QD-based light-emitting diodes (QLEDs) in 1994, several QDs such as CdS, CdTe, PbSe, PbS, and InP@ZnSeS have been produced as potential materials for QLEDs, solar cells, display devices, photodetectors, fluorescent probes, and various biomedical diagnostics [5,6,7,8]. However, some drawbacks such as self-aggregation, toxicity, and heavy metal content are mostly limiting their practical applications [9].

In many regions of the world, the use of heavy metals in many household goods is restricted, and thus, most of the cadmium-based quantum dots are unfeasible for indoor applications [10,11]. In addition, the studies on QD toxicity have focused on cadmium and lead-containing particles, as CdSe QDs release free cadmium when irradiated with ultraviolet (UV) light or oxidized by air [12,13,14]. In the absence of UV light, QDs coated with a stable polymer are essentially non-toxic. However, though surface coatings like ZnS and bovine serum albumin (BSA), which are used to reduce surface oxidation by preventing the self-aggregation of QDs and enhance quantum yield and stability, reduce concerns of toxicity, they do not wholly eliminate the cytotoxicity [15,16], and thus there is a need for the development of non-toxic and cost effective QDs.

In order to address this toxicity issue, we selected self-activated CsVO_3_ as a host material in which vanadium exists in the ‘5+’ oxidation state, as in this form vanadium is non-toxic, stable, and cheaper than cadmium and lead-containing raw materials. In addition, the vanadate cluster [VO_4_]^3−^, in which the central metal ion is coordinated by four oxygen ions in tetrahedral (T_d_) symmetry, serves as an efficient luminescent center and exhibits excellent quantum efficiency [17,18,19]. From the biological viewpoint, only the higher oxidation states (4+ and 5+) of vanadium are given importance in biomedical research due to the ability of vanadium in these states to participate in redox reactions when coordinated with its ligands, which is a remarkable property well utilized in drug design. Furthermore, vanadate has numerous biological activities, not the least of which is its ability to inhibit many enzymes [20,21]. Therefore, to avoid the above-mentioned limitations of Cd and Pb-containing QDs, we undertook the development of CsVO_3_ QDs for various optoelectronic and biomedical applications.

In this study, we synthesized novel cadmium-free CsVO_3_ QDs using a simple hotplate-based method for the first time. The sizes of the CsVO_3_ QDs produced were tuned from 2 to 10 nm by varying the reaction temperature from 140 to 190 °C. Band gap energies were calculated from absorption spectra. The existence of the quantum confinement effect was established using absorption and photoluminescence (PL) studies. In addition, CsVO_3_ nanosheets self-assembled microflower-like particles were obtained as residue after centrifuging the reaction mixture. Morphological studies were conducted on these nanosheets using scanning and transmission electron microscopy (SEM and TEM), and their crystalline nature was examined by energy-dispersive X-ray spectroscopy (EDX) and X-ray diffraction (XRD). The PL properties of CsVO_3_ QDs and the microflower-like particles were studied using an excitation wavelength of 365 nm.

## 2. Materials and Methods

### 2.1. Materials

Cesium nitrate [CsNO_3_] (Sigma-Aldrich, Seoul, South Korea; high purity grade), ammonium metavanadate [NH_4_VO_3_] (Sigma-Aldrich, high purity grade), 1-octadecene [CH_3_(CH_2_)_15_CH=CH_2_] (Sigma-Aldrich; technical grade, 90%), oleylamine [CH_3_(CH_2_)_7_CH=CH(CH_2_)_7_CH_2_NH_2_] (Sigma-Aldrich; technical grade, 70%), oleic acid [CH_3_(CH_2_)_7_CH=CH(CH_2_)_7_COOH] (Daejung, Sinan, South Korea; extra pure), ammonium hydroxide solution [NH_4_OH] (Sigma-Aldrich; ~25% NH_3_ basis), and acetone [CH_3_COCH_3_] were of analytical grade. Triple-distilled de-ionized (DI) water was used to dissolve the CsNO_3_. All the above-mentioned reagents were used without additional purification.

### 2.2. Synthesis of CsVO_3_ QDs

The required amount of CsNO_3_ was dissolved in 10 mL of DI water in a beaker. Separately, NH_4_VO_3_ was dissolved in a solvent mixture (15 mL octadecene, 3 mL oleylamine, and 1.5 mL oleic acid) in another beaker, placed on a hot plate set at 50 °C, and stirred for 1 h. The Cs solution was added dropwise under continuous magnetic stirring, and then the hot plate temperature was increased to 170 °C. After 1 h, 1 mL of NH_4_OH was added to the mixture and stirred for 2 h. The reaction mixture was then cooled to room temperature, and 20 mL of acetone was added to precipitate the CsVO_3_ QDs. The CsVO_3_ nanosheets self-assembled microflower-like particles were obtained by centrifugation at 10,000 rpm for 15 min. The residue was again washed with acetone and then dried under ambient conditions for 8 h. The experiment was repeated at different reaction temperatures of 140, 150, 160, 180, and 190 °C to achieve color tunability of CsVO_3_ QDs. Details of the characterization techniques used are provided in the Supplementary Materials.

## 3. Results and Discussion

**Figure 1:**
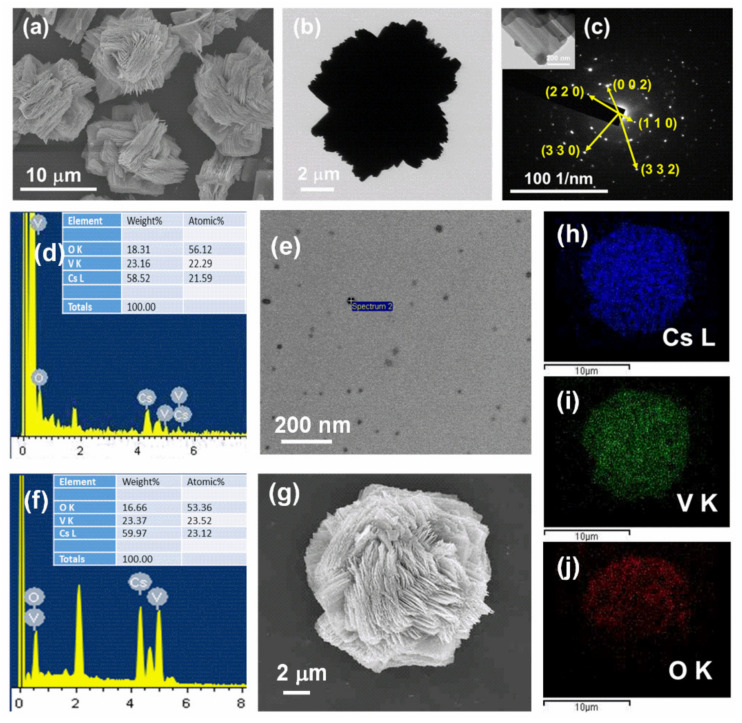
(**a**–**c**) SEM and TEM images and SAED pattern of CsVO_3_ nanosheets self-assembled microflower-like particles. (**d**,**e**) EDX spectrum and the corresponding TEM image of CsVO_3_ QDs. (**f**–**j**) EDS spectrum, SEM image, and the elemental mapping of a CsVO_3_ microflower-like particle.

Highly stable and cost-effective CsVO_3_ QDs were produced by facile hotplate synthesis. The spherical-shaped CsVO_3_ QDs were precipitated using acetone and separated from the solvent (as supernatant) after centrifugation for 15 min at 10,000 rpm. The CsVO_3_ nanosheets self-assembled microflower-like particles were found as residue after centrifugation and the resultant SEM and TEM images are presented in Figure 1a,b. The selected area (electron) diffraction pattern of CsVO_3_ nanosheets displayed a ring pattern with bright spots overlaid on it (Figure 1c), indicating the nanocrystalline nature of the particles. The obtained d-spacings of 2.87, 4.93, 1.43, 1.64, and 2.45 corresponded to the (0 0 2), (1 1 0), (3 3 2), (3 3 0), and (2 2 0) planes of orthorhombic CsVO_3_ matrix (JCPDS No. 070-0680). The chemical compositions of CsVO_3_ QDs and the microflower-like particles (synthesized at 170 °C) were examined by EDX analysis; the resultant EDX spectra of CsVO_3_ QDs and the microflower-like particles are presented in Figure 1d–g. The atomic and weight percentages of Cs, V, and O elements in CsVO_3_ QDs and microflower-like particles are displayed as insets in Figure 1d,f, respectively. The position of X-ray peaks in both spectra showed ‘V’ and ‘O’ ions occupied the K-shell, and that ‘Cs’ ions occupied the L-shell of the energy spectrum at 4.949, 0.525, and 4.286 eV, respectively. Elemental mapping of the microflower-like particle confirmed that Cs, V, and O were homogeneously distributed within the particle (Figure 1h–j).

**Figure 2:**
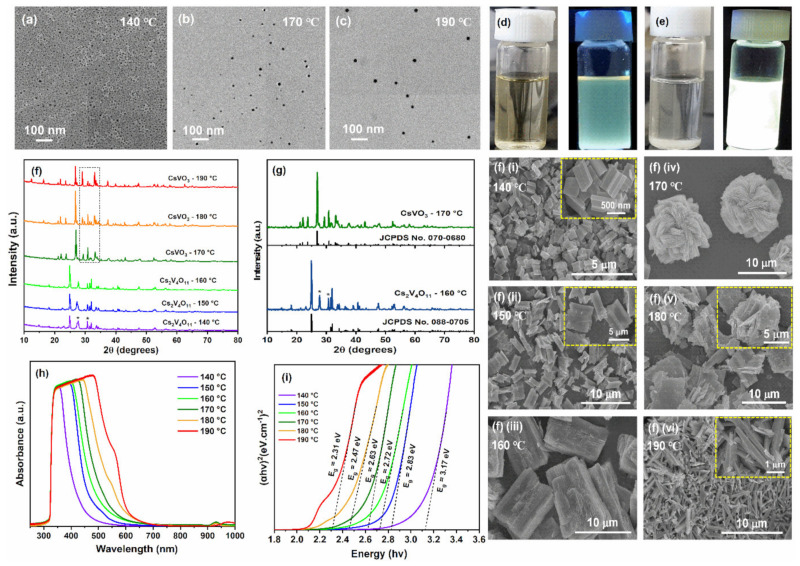
(**a**–**c**) TEM images of CsVO_3_ QDs synthesized at 140, 170, and 190 °C, respectively. (**d**,**e**) Digital photos of QDs and microflower-like particles under day light and UV light. (**f**,**g**) XRD patterns of CsVO_3_ powder samples at different reaction temperatures and (**f**)(i–vi) the corresponding SEM images of the CsVO_3_ samples. (**h**,**i**) Absorption spectra and corresponding Tauc plots of CsVO_3_ QDs synthesized at different temperatures.

The TEM images of CsVO_3_ QDs synthesized at 140 °C (blue), 170 °C (green), and 190 °C (red) are shown in Figure 2a–c, and TEM images of CsVO_3_ QDs synthesized at 150 °C (sky blue), 160 °C (cyan), and 180 °C (yellow) are presented in Figure S1. The size of the QDs increased monotonically by increasing the reaction temperature from 140 to 190 °C as shown in Figure 2a–c, confirming the existence of the quantum confinement effect. According to the literature, the quantum confinement effect alters the optical and electrical properties of semiconductor nanocrystals (2 to 10 nm), and causes size-dependent changes in fluorescence wavelengths [1]. At a reaction temperature of 140 °C, particle sizes of 2–3 nm were obtained, which fluoresced blue, whereas at temperatures of 170 and 190 °C, CsVO_3_ QDs with particle sizes of 5–6 and 8–10 nm were obtained that fluoresced green and red, respectively. A plot of emission versus size of CsVO_3_ QDs was presented in supplementary information Figure S2. Digital photographs of CsVO_3_ QDs and the microflower-like particles (synthesized at 170 °C) under daylight and UV-light (365 nm) are shown in Figure 2d,e, respectively. As can be seen from the figure, the CsVO_3_ QDs and microflower-like particles exhibited green and white emission, respectively. In order to determine the crystalline phase of QDs, the XRD patterns were recorded for the corresponding powder samples. Figure 2f shows the XRD patterns of CsVO_3_ microflower-like particles at reaction temperatures from 140 to 190 °C; corresponding SEM images are presented in Figure 2f(i–vi), respectively. Up to 160 °C, the obtained diffraction patterns were indexed to the orthorhombic phase of Cs_2_V_4_O_11_ with space group Cmm2 (JCPDS No. 088-0705) and some cesium impurity peaks, whereas the XRD patterns of samples synthesized at 170, 180, and 190 °C were well matched with the orthorhombic phase of CsVO_3_ with space group Pbcm(57) (JCPDS No. 070-0680). Figure 2g shows the reference patterns of Cs_2_V_4_O_11_ and CsVO_3_ and the diffraction patterns obtained for CsVO_3_ powder samples synthesized at 160 and 170 °C. On raising the reaction temperature from 170 to 190 °C, the relative intensities of XRD peaks located at 29°, 30°, and 33° corresponding to the (0 4 0), (0 0 2) and (0 4 1) planes altered (Figure 2f), which was attributed to a change in nanocrystal orientation, as it is well-established that the preferred orientations depend on the nanoparticle size and shape [17,22]. As can be seen from the figure, the nanosheets that self-assembled as microflower-like particles (Figure 2f(iv)) became rod-like particles (Figure 2f(vi)) on increasing the reaction temperature from 170 to 190 °C, and the preferred orientation of rod-like particles is mostly in one direction. Therefore, as a result of preferential orientation, the intensities of (0 4 0), (0 0 2), and (0 4 1) planes altered in the XRD pattern. Furthermore, the average crystallite size (D) of the microflower-like particles was calculated using the well-known Scherrerer equation (D_hkl_ = κλ/β cosθ, [23,24] where k is scherrer constant, λ is x-ray beam wavelength, θ is diffraction angle, and β represents the full width at half maximum of the diffraction peak. The average crystallite size was calculated to be 57.6 nm.

**Figure 3:**
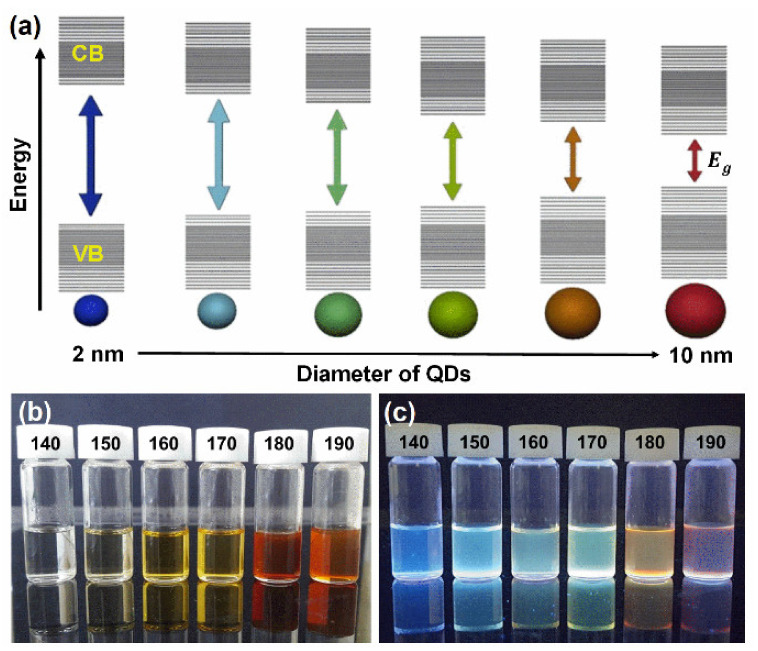
(**a**) Schematic illustration of quantum confinement effect in CsVO_3_ QDs, (**b**,**c**) digital photographs of CsVO_3_ QDs synthesized at different reaction temperatures in the absence and presence of UV light.

The absorption spectra of CsVO_3_ QDs synthesized at different reaction temperatures are presented in Figure 2h. All spectra displayed a strong absorption band between 320 to 620 nm, which suggested that the CsVO_3_ QDs are suitable for near-UV and visible excitation-based QLEDs. As the reaction temperature was increased from 140 to 190 °C, the broadness of the absorption band increased and the band maxima redshifted from 354 to 480 nm, which confirmed an increase in CsVO_3_ QD size. The optical band gap energies (*E_g_*) of CsVO_3_ QDs synthesized at different reaction temperatures were estimated using Tauc’s relation [25,26]:

(1) $$\alpha hv=B{\left(hv-E_{g}\right)}^{n}$$

where α represents the absorption coefficient, B is the proportionality constant, photon energy is represented by hv, and n adopts values 1/2, 2, 3/2, or 3 depending on the type of transition. According to Tauc’s relation, n takes the value of 1/2 and 2 for direct and indirect allowed transitions, whereas the value of n becomes 3/2 and 3 for direct and indirect forbidden transitions, respectively. Straight line plots were obtained for ${\left(\alpha hv\right)}^{2}$ versus $\left(hv\right)$ signifying the direct allowed transitions. The *E_g_* values of CsVO_3_ QDs were evaluated by extrapolating the linear regions onto the energy axis, as shown in Figure 2i. Estimated *E_g_* values of CsVO_3_ QDs synthesized at temperatures of 140, 150, 160, 170, 180, and 190 °C were 3.17, 2.83, 2.72, 2.63, 2.47, and 2.31 eV, respectively. As expected, *E_g_* values decreased with increasing QD size, which confirmed the presence of quantum confinement effect. For CsVO_3_ QDs, the band gap of 2–3 nm sized QDs was 3.17 eV, whereas the band gap of 8–10 nm sized QDs was 2.31 eV, which means the red QDs (8–10 nm) need less energy than blue QDs (2–3 nm) to reach the conduction band and thus release less energy when they return to the ground state. A schematic illustration of the quantum confinement effect is provided in Figure 3a. Digital photographs of CsVO_3_ QDs in the absence and presence of UV-light are shown in Figure 3b,c, respectively.

**Figure 4:**
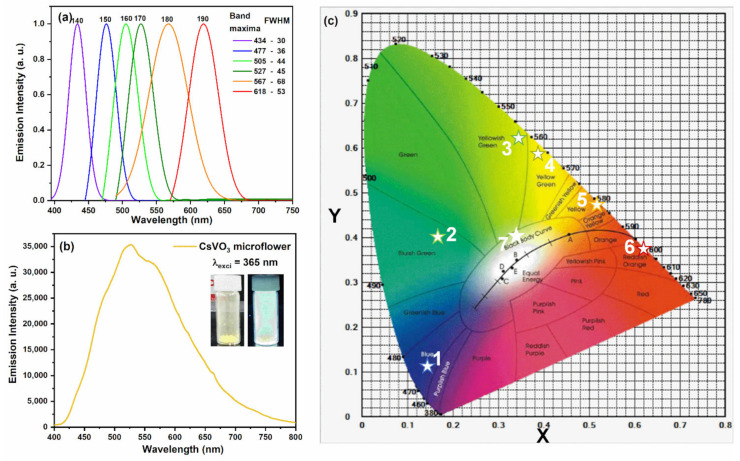
(**a**) Luminescence spectra of CsVO_3_ QDs synthesized at different reaction temperatures from 140 to 190 °C, (**b**) PL emission spectrum of microflower-like CsVO_3_ particles excited at 365 nm, and (**c**) CIE chromaticity coordinates of CsVO_3_ QDs and microflower-like particles ((1–6) CsVO_3_ QDs and (7) CsVO_3_ microflower-like particles).

Figure 4a shows the PL spectra of CsVO_3_ QDs synthesized at different reaction temperatures. As the reaction temperature increased from 140 to 190 °C, the emission band maxima shifted from 434 to 618 nm, as shown in Figure 4a. Usually, the emission wavelength of QDs is proportional to its size, and hence the emission wavelengths are redshifted along with the growing size of CsVO_3_ QDs [27]. Full width at half maxima (FWHMs) of emission spectra were found to be 30, 36, 44, 45, 68, and 53 nm for CsVO_3_ QDs synthesized at 140 (blue), 150 (sky blue), 160 (cyan), 170 (green), 180 (yellow), and 190 °C (red), respectively. The narrow emission bands of blue and green QDs and lower FWHM values demonstrated a homogeneous distribution of CsVO_3_ QDs, whereas higher FWHM values indicated that the yellow and red QDs were not uniform in size. Figure 4b shows the PL emission spectrum of CsVO_3_ microflower-like particles excited at 365 nm. The acquired emission spectrum covered the whole visible area (between 420 and 750 nm) with a band maximum at 526 nm, as shown in Figure 4b. Usually, the transfer of charge from a 2p orbital of $O^{2-}$ to a 3d orbital of $V^{5+}$ in $VO_{4}^{3-}$ group with tetrahedral symmetry leads to the broad band emission of self-activated vanadate phosphors [28], which is perfectly suitable for near-UV based white-LEDs (WLEDs). These CsVO_3_ microflower-like particles could potentially replace the conventional rare earth-based WLEDs in the lighting industry.

Commission International De I’Eclairage (CIE) coordinates were calculated for different colored CsVO_3_ QDs and are displayed in Figure 4c. All QDs have excellent chromaticity coordinates in their respective emission regions. CsVO_3_ QDs synthesized at 140, 150, 160, 170, 180, and 190 °C had CIE chromaticity coordinates in the blue (0.148, 0.112), bluish green (0.166, 0.402), yellowish green (0.342, 0.622), yellow green (0.387, 0.584), yellow (0.522, 0.472), and reddish orange (0.623, 0.376) regions, respectively. On the other hand, CsVO_3_ microflower-like particles showed excellent chromaticity coordinates in the white emission region (0.341, 0.402). Furthermore, the acquired CIE coordinates of blue and red QDs were close to the National Television System Committee (NTSC colorimetry (1953)) approved standard blue (0.14, 0.08) and red (0.67, 0.33) coordinates. Nonetheless, further studies are required to optimize the performances of the CsVO_3_ QDs, whereas microflower-like CsVO_3_ appear to be eminently suitable for use in solid-state lighting applications.

## 4. Conclusions

In this study, we synthesized novel cadmium-free CsVO_3_ QDs by facile hotplate synthesis for the first time. The sizes of the QDs were controlled from 2 to 10 nm by increasing the reaction temperature from 140 to 190 °C, which resulted in a band gap reduction from 3.17 to 2.31 eV and red-shifting of the PL emission spectra, demonstrating the presence of quantum confinement effect. Estimated FWHM values indicated that blue and green QDs were more homogeneous than yellow and red QDs. Furthermore, the CsVO_3_ nanosheets self-assembled microflower-like particles were obtained as residue at a reaction temperature of 170 °C. The obtained (as-synthesized) microflower-like particles were well crystallized in the orthorhombic phase and exhibited broad emission from 420 to 750 nm under 365 nm excitation. The calculated CIE coordinates of blue and red QDs were close to the NTSC colorimetry (1953) approved standard blue and red color coordinates, and CIE coordinates of the CsVO_3_ microflower-like particles were in the warm white emission region.

## Data Availability

The data that support the findings of this study are available from the corresponding authors upon reasonable request.

## Supplementary Materials

The following supporting information can be downloaded at: https://www.mdpi.com/article/10.3390/nano12162864/s1, Figure S1: TEM images of CsVO_3_ QDs synthesized at 150, 160, and 180 °C, and the video clip of CsVO_3_ QDs in the absence and presence of UV light, Figure S2: Emission wavelength versus CsVO_3_ QDs size.
