# Supplementary material for: A Novel and Cost-Effective CsVO3 Quantum Dots for Optoelectronic and Display Applications
Source: Nanomaterials (Basel). 2022 Aug 19;12(16):2864. doi: 10.3390/nano12162864 (PMC9412482; doi:10.3390/nano12162864)
Supplement: Supplementary file 1 [file nanomaterials-12-02864-s001.zip › nanomaterials-1853521-supplementary.pdf]

nanomaterials

Supplementary Materials

A Novel and Cost-Effective $CsVO_3$ Quantum Dots for Optoelectronic and Display Applications

Ganji Seeta Rama Raju [1], Ganji Lakshmi Varaprasad [2], Jeong-Hwan Lee [3], Jin Young Park [4], Nilesh R. Chodankar [1], Kugalur Shanmugam Ranjith [1], Eluri Pavitra [2,*], Yun Suk Huh [2,*] and Young-Kyu Han [1,*]

1 Department of Energy and Materials Engineering, Dongguk University-Seoul, Seoul 04620, Korea
2 Department of Biological Engineering, Biohybrid Systems Research Center (BSRC), Inha University, Incheon 22212, Korea
3 Department of Materials Science and Engineering, Inha University, Incheon 22212, Korea
4 Department of Electrical, Electronics and Software Engineering, Pukyong National University, Yongdang Campus, Busan 48547, Korea
* Correspondence: pavitra@inha.ac.kr (E.P.); yunsuk.huh@inha.ac.kr (Y.S.H.); ykenergy@dongguk.edu (Y.-K.H.)

Characterizations

The morphology of the $CsVO_3$ QDs and $CsVO_3$ nanosheets self-assembled micro-flower-like particles were examined using field-emission scanning electron microscopy (FE-SEM) (Hitachi-SU8010, Japan) and field-emission transmission electron microscopy (FE-TEM: JEM-2100F/JEOL) measurements. The X-ray diffraction (XRD) patterns of $CsVO_3$ microflower-like particles were measured using X'Pert Pro multipurpose X-ray diffractometer (PANalytical) with ceramic Cu target (CuKα= 1.5406 Å), X-ray generator of 3kW 60kV/60mA, and scan speed of 1 deg/sec. The energy dispersive X-ray spectrometer attached to the FE-SEM instrument (PANalytical, Holland) was used to verify the elemental composition of $CsVO_3$ QDs and microflower-like particles. The photoluminescence emission spectrum of $CsVO_3$ microflower-like particles was measured using a Photon Technology International (PTI, USA) fluorimeter with a xenon arc lamp power of 60 W. The absorption spectra of $CsVO_3$ QDs were recorded on a V-770 UV-vis spectrophotometer (JASCO International Co. Ltd., Japan).

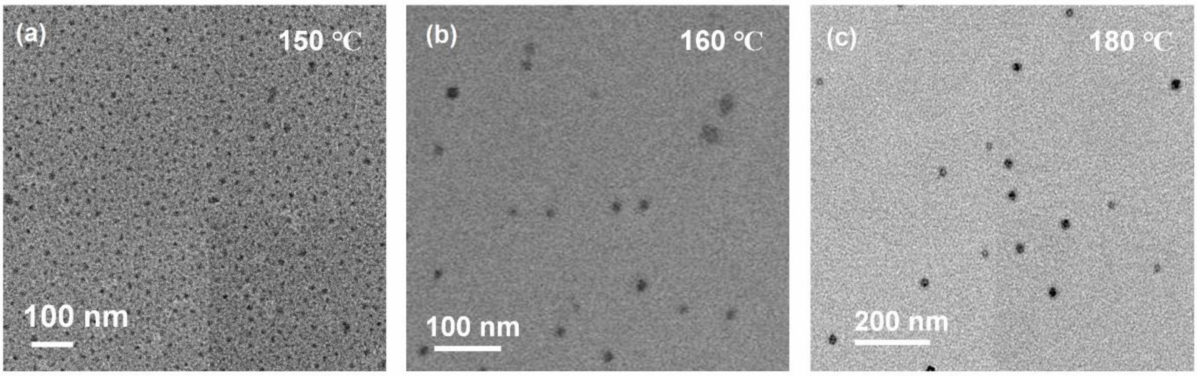

Figure S1. (**a–c**) FE-TEM images of the $CsVO_3$ QDs synthesized at 150 °C, 160 °C, and 180 °C, respectively.

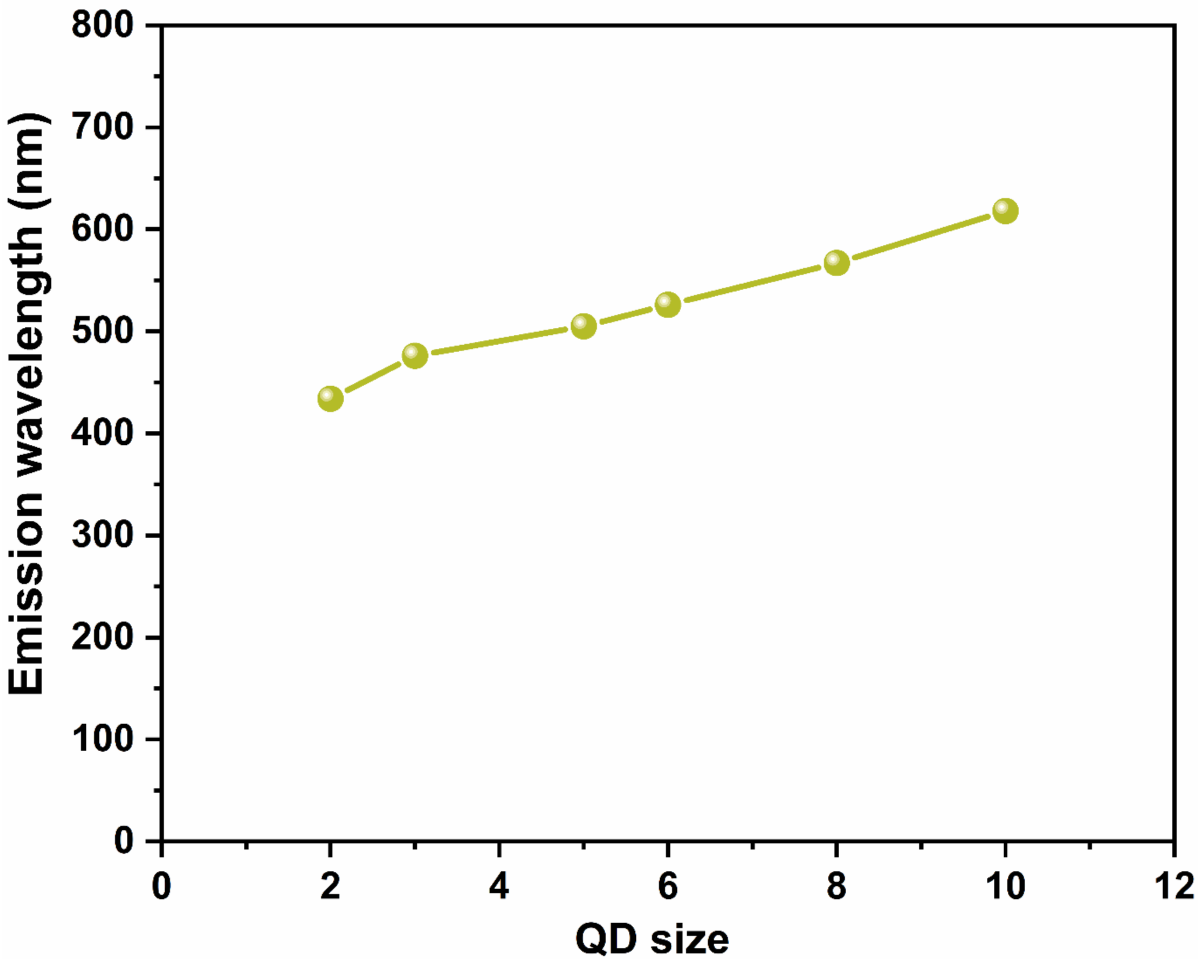

Figure S2. Emission wavelength versus CsVO3 QDs size.
